# Using multimodal MRI to investigate alterations in brain structure and function in the BBZDR/Wor rat model of type 2 diabetes

**DOI:** 10.1002/ame2.12140

**Published:** 2020-12-29

**Authors:** Christopher M. Lawson, Kilian F. G. Rentrup, Xuezhu Cai, Praveen P. Kulkarni, Craig F. Ferris

**Affiliations:** ^1^ Center for Translational NeuroImaging Northeastern University Boston MA USA

**Keywords:** BBZDR/Wor rat, diffusion weighted imaging, magnetic resonance imaging, resting‐state BOLD functional imaging, small vessel disease, voxel‐based morphometry

## Abstract

**Background:**

This is an exploratory study using multimodal magnetic resonance imaging (MRI) to interrogate the brain of rats with type 2 diabetes (T2DM) as compared to controls. It was hypothesized there would be changes in brain structure and function that reflected the human disorder, thus providing a model system by which to follow disease progression with noninvasive MRI.

**Methods:**

The transgenic BBZDR/Wor rat, an animal model of T2MD, and age‐matched controls were studied for changes in brain structure using voxel‐based morphometry, alteration in white and gray matter microarchitecture using diffusion weighted imaging with indices of anisotropy, and functional coupling using resting‐state BOLD functional connectivity. Images from each modality were registered to, and analyzed, using a 3D MRI rat atlas providing site‐specific data on over 168 different brain areas.

**Results:**

There was an overall reduction in brain volume focused primarily on the somatosensory cortex, cerebellum, and white matter tracts. The putative changes in white and gray matter microarchitecture were pervasive affecting much of the brain and not localized to any region. There was a general increase in connectivity in T2DM rats as compared to controls. The cerebellum presented with strong functional coupling to pons and brainstem in T2DM rats but negative connectivity to hippocampus.

**Conclusion:**

The neuroradiological measures collected in BBBKZ/Wor rats using multimodal imaging methods did not reflect those reported for T2DB patients in the clinic. The data would suggest the BBBKZ/Wor rat is not an appropriate imaging model for T2DM.

## 1 INTRODUCTION

Diabetes is a serious metabolic disorder estimated to affect 30 million people in the United States as of 2016, with prevalence expected to reach more than 54.9 million Americans by 2030.^41^, ^43^ Diabetes is broken down into two main categories, type 1 diabetes (T1DM) and type 2 diabetes (T2DM). Destruction of pancreatic beta cells resulting in insulin deficiency is the hallmark of T1DM while T2DM is typically the result of a combination of peripheral insulin resistance and dysfunctional insulin secretion by pancreatic beta cells.^39^ T2DM is much more common in the United States with 90%‐95% of all diabetes cases being such.^41^ The pathology of T2MD is systemic, affecting much of the body and most functions. The brain is not spared as there are severe effects on cognition and behavior with disease progression and aging.^3^ Studies with magnetic resonance imaging (MRI) report abnormalities in cerebral macrostructure and microstructure such as cortical atrophy,^46^ regional reductions in brain volume,^34^ structural deformities in cerebral gray matter,^59^ increased white matter lesions^2^, ^36^ and changes in blood‐brain barrier permeability.^49^ Indeed, there is a large body of literature using multimodal MRI eg voxel based morphometry (VBM), diffusion weighted imaging (DWI) and resting‐state BOLD functional MRI (rsFC), to interrogate brain structure and function in T2DM patients to better understand disease progression and prognosis for cognitive decline (for review see Ref.[4]).

There is a paucity of MRI studies in animal models of T2DM using imaging modalities commonly performed in the clinic. We know of only two such studies, one looking at ischemic vascular damage and axonal density following stroke in the high‐fat diet, streptozotocin‐treated Wistar rat (HFD/STZ),^7^ and a second in the TALLYHO/JngJ (TH) mouse correlating white matter connectivity using DWI with compulsive behavior.^53^ To address this shortcoming, we performed an exploratory study using VBM, DWI, and rsFC to interrogate the brain of the obese Bio‐Breeding Zucker diabetic (BBZDR/Wor) rat, a model of T2DM.^52^ Our findings are discussed in the context of their clinical relevance and whether this animal model and the imaging modalities used would have translational value in a larger prospective study following evolution of cerebral neuropathy in T2DM.

## 2 METHODS

### 2.1 Animal model

Male Bio‐Breeding Zucker diabetic rats (BBZDR/Wor rats) (n = 8) as well as age‐matched nondiabetic BBZDR littermates (n = 8), were obtained from Biomere in Worcester, MA for imaging. The company decided to retire the breeding line and made a gift of their last animals to the Center for Translational NeuroImaging. The BBZDR/Wor rat is an inbred rat strain of T2DM developed by crossing the BBDP/Wor rat, a lean, lymphopenic rat with autoimmune, insulin‐dependent diabetes mellitus, with the Zucker fatty rat, an obese rat with insulin resistance and glucose intolerance. In BBZDR/Wor animals, the recessive Iddm2 gene responsible for lymphopenia and spontaneous autoimmunity is removed while the Lepr fa (fa1) mutation is retained. Obese male BBZDR/Wor rat spontaneously develops type 2 diabetes at approximately 10 weeks of age (~100%) when fed standard rat chow.^8^, ^9^, ^10^, ^52^ The BBZDR/Wor diabetic rat displays the clinical symptoms typically associated with T2DM including dyslipidemia, hyperglycemia, insulin resistance, hypertension, and decreased levels of the beta cell‐specific glucose transporter type‐2 (GLUT‐2).^8^, ^47^, ^52^

Rats were maintained on a 12:12 hours light‐dark cycle with lights on at 07:00 hours, allowed access to food and water ad libitum and were treated with IP injections of saline at indications of weight loss. The average age of the animals at time of imaging was about 4 months. All animal experiments were conducted in accordance with the Northeastern University Division of Laboratory Animal Medicine and Institutional Animal Care and Use Committee and adhere to the ARRIVE guidelines for reporting in vivo experiments in animal research^29^

### 2.2 Neuroimaging

Imaging was conducted using a Bruker Biospec 7.0T/20‐cm USR horizontal magnet (Bruker, Billerica, MA, USA) and a 20‐G/cm magnetic field gradient insert (ID = 12 cm) capable of a 120‐μs rise time. Radiofrequency signals were sent and received with a quadrature volume coil built into the animal restrainer (Animal Imaging Research, Holden, Massachusetts). All rats were restrained using a custom restraint kit and imaged under 1%‐2% isoflurane while keeping a respiratory rate of 40‐50/min.

### 2.3 Voxel‐based morphometry

Images were acquired using RARE sequence with TR/TE = 3310/36ms; matrix size 256 × 256 × 40, field of view = 30 × 30 mm, spatial resolution, 0.117 × 0.117 × 0.7 mm. A 3D MRI Rat Brain Atlas (© 2012 Ekam Solutions LLC, Boston, MA) was used to calculate brain volumes, and registered the standard structural rat template image onto high resolution T2‐weighted images for each subject using a nonlinear registration method implemented by Unix based software package Deformable Registration via Attribute Matching and Mutual‐Saliency Weighting (DRAMMS; https://www.cbica.upenn.edu/sbia/software/dramms/index.html). The atlas (image size 256x256x63) (H × W × D) was then warped from the standard space into the subject image space (image size 256 × 256 × 40) using the deformation obtained from the above step using the nearest‐neighbor interpolation method. In the volumetric analysis, each brain region was therefore segmented, and the volume values were extracted for all 171 ROIs, calculated by multiplying unit volume of voxel (in mm^3^) by the number of voxels using an in‐house MATLAB script. To account for different brain sizes, all ROI volumes were normalized by dividing each subject's ROI volume by their total brain volume.

### 2.4 Diffusion weighted imaging—quantitative anisotropy

DWI was acquired with a spin‐echo echo‐planar‐imaging (EPI) pulse sequence having the following parameters: TR/TE = 500/20 msec, eight EPI segments, 10 noncollinear gradient directions with a single b‐value shell at 1000 s/mm 2 and one image with a B‐value of 0 s/mm 2 (referred to as B 0), gradient duration (δ): 4.5 msec separated by time interval (Δ): 8.7 msec, time for one average: 24.76 min, 2 averages (to reduce noise): 49.52 min. Geometrical parameters were: 48 coronal slices, each 0.313 mm thick (brain volume) and with in‐plane resolution of 0.313 × 0.313 mm 2 (matrix size 96 × 96; FOV 30 mm^3^). Each DWI acquisition took 35 minutes and the entire MRI protocol lasted about 70 minutes. Image analysis included DWI analysis of the DW‐3D‐EPI images to produce the maps of fractional anisotropy (FA), apparent diffusion coefficient (ADC), axial diffusivity (AD), and radial diffusivity (RD). DWI analysis was completed with MATLAB and MedINRIA (1.9.0; http://www‐sop.inria.fr/asclepios/ software/MedINRIA/index.php) software. Because sporadic excessive breathing during DWI acquisition can lead to significant image motion artifacts that are apparent only in the slices sampled when motion occurred, each image (for each slice and each gradient direction) was screened, prior to DWI analysis. If found, acquisition points with motion artifacts were eliminated from analyses.

For statistical comparisons between rats, each brain volume was registered to the 3D rat atlas allowing voxel‐ and region‐based statistics. All image transformations and statistical analyses were carried out using the in‐house MIVA software (http://ccni.wpi.edu/). For each rat, the B 0 image was co‐registered with the B 0 template (using a 6‐parameter rigid‐body transformation). The co‐registration parameters were then applied on the DWI indexed maps for the different indices of anisotropy. Normalization was performed on the maps since they provided the most detailed visualization of brain structures and allow for more accurate normalization. The normalization parameters were then applied to all DWI indexed maps that were then smoothed with a 0.3‐mm Gaussian kernel. To ensure that FA and ADC values were not affected significantly by the preprocessing steps, the ‘nearest neighbor’ option was used following registration and normalization.

Statistical differences in measures of DWI between experimental groups were determined using a nonparametric Mann‐Whitney *U* test (alpha set at 5%). The formula below was used to account for false discovery from multiple comparisons.

$$P\left(i\right)\leq \frac{i}{V}\frac{q}{c\left(V\right)}$$

*P*(*i*) is the *P*‐value based on the *t* test analysis. Each of 171 ROIs (*i*) within the brain containing (*V*) ROIs was ranked in order of its probability value (see Table 1). The false‐positive filter value *q* was set to 0.2 and the predetermined *c*(*V*) was set to unity11. The corrected probability is noted on each table.

**Table 1:** Apparent diffusion coefficient

Apparent diffusion coefficient							
Brain Area	Control			BBDRZ/Wor			
	Ave	SD		Ave	SD	*P*‐val	Ω sq
Basal amygdaloid n.	2.13	0.14	>	1.87	0.07	.002	0.722
Lateral amygdaloid n.	2.20	0.14	>	1.83	0.12	.002	0.722
Pontine reticular n. caudal	2.54	0.29	>	2.05	0.19	.002	0.722
Parvicellular reticular n.	2.62	0.25	>	1.83	0.39	.002	0.720
Caudal piriform ctx	2.18	0.12	>	1.61	0.13	.002	0.720
Gigantocellularis reticular n.	2.79	0.28	>	2.03	0.42	.002	0.719
Retrosplenial rostral ctx	2.55	0.22	>	2.09	0.15	.002	0.719
Ventral subiculum	2.42	0.29	>	1.95	0.13	.002	0.719
Cortical amygdaloid n.	2.42	0.22	>	1.84	0.22	.002	0.688
Frontal association ctx	2.59	0.15	>	1.78	0.28	.003	0.655
Habenula n.	3.03	0.21	>	2.37	0.30	.003	0.653
Root of trigeminal nerve	3.12	0.26	>	2.25	0.48	.003	0.651
Anterior lobe pituitary	2.97	0.49	>	1.60	0.72	.003	0.651
CA3 hippocampus ventral	2.29	0.18	>	1.94	0.11	.003	0.651
Crus 1 of ansiform lobule	2.19	0.34	>	1.48	0.28	.003	0.651
Entorhinal ctx	2.68	0.26	>	1.97	0.20	.003	0.651
Glomerular layer	2.66	0.12	>	1.87	0.44	.003	0.651
Paraflocculus cerebellum	2.29	0.18	>	1.86	0.22	.003	0.651
Solitary tract n.	2.91	0.45	>	1.97	0.36	.003	0.651
Superior colliculus	2.57	0.23	>	2.15	0.12	.004	0.591
Dorsomedial tegmental area	2.57	0.29	>	2.15	0.13	.004	0.589
Medial amygdaloid n.	2.84	0.21	>	2.41	0.18	.004	0.588
Reticular n.	2.06	0.08	>	1.87	0.11	.005	0.558
CA1 hippocampus ventral	2.14	0.21	>	1.77	0.19	.006	0.530
5th cerebellar lobule	2.85	0.29	>	2.16	0.30	.006	0.529
Inferior colliculus	2.87	0.32	>	2.39	0.18	.006	0.529
Visual 1 ctx	2.30	0.31	>	1.92	0.16	.006	0.529
Central amygdaloid n.	2.42	0.23	>	1.99	0.17	.006	0.527
Dentate gyrus ventral	2.67	0.35	>	2.13	0.16	.006	0.527
Vestibular n.	2.86	0.38	>	2.21	0.25	.006	0.527
Medial dorsal thalamic n.	2.18	0.15	>	1.90	0.18	.007	0.501

### 2.5 Resting‐state functional connectivity

Scans were collected using a spin‐echo triple‐shot EPI sequence (imaging parameters: matrix size = 96x96x20 (H x W x D), TR/TE = 1000/15 msec, voxel size = 0.312 x 0.312 x 1.2 mm, slice thickness = 1.2 mm, with 200 repetitions, time of acquisition 10 min). There are numerous studies detailing the benefits of multi‐shot EPI in BOLD imaging.^20^, ^26^, ^37^, ^40^, ^50^ We avoided using single‐shot EPI because of its severe geometrical distortion at high field strengths (≥7T) and loss of effective spatial resolution as the readout period increases.^12^, ^20^, ^23^ There is also the possibility of signal loss in single shot EPI due to accumulated magnetic susceptibility or field inhomogeneity.^26^

Preprocessing in this study was accomplished by combining Analysis of Functional NeuroImages (AFNI_17.1.12, http://afni.nimh.nih.gov/afni/), FMRIB Software library (FSL, v5.0.9, http://fsl.fmrib.ox.ac.uk/fsl/), Deformable Registration via Attribute Matching and Mutual‐Saliency Weighting (DRAMMS 1.4.1,https://www.cbica.upenn.edu/sbia/software/dramms /index.html), and MATLAB (Mathworks, Natick, MA). Brain tissue masks for resting‐state functional images were manually drawn using a 3DSlicer (https://www.slicer.org/) and applied for skull stripping. Motion outliers (ie, data corrupted by extensive motion) were detected in the dataset and the corresponding time points were recorded so that they could be regressed out in a later step. Functional data were assessed for the presence of motion spikes. Any large motion spikes were identified and removed from the time‐course signals. This filtering step was followed by slice timing correction from interleaved slice acquisition order. Head motion correction (six motion parameters) was carried out using the first volume as a reference image. Normalization was completed by registering functional data to the 3D MRI Rat Brain Atlas (© 2012 Ekam Solutions LLC, Boston, MA) using affine registration through DRAMMS. The 3D MRI Rat Brain Atlas containing 171 annotated brain regions was used for segmentation. Data are reported in 166 brain areas, as 5 regions in the brain atlas were excluded from analysis due to the large size of three brains. These brains fell slightly outside our imaging field of view and thus we did not get any signal from the extreme caudal tip of the cerebellum. Whole brains that contain all regions of interest are needed for analyses so rather than excluding the animals, we removed the brain sites across all animals. After quality assurance, band‐pass filtering (0.01‐0.1 Hz) was performed to reduce low‐frequency drift effects and high‐frequency physiological noise for each subject. The resulting images were further detrended and spatially smoothed (full width at half maximum = 0.8 mm). Finally, regressors comprised of motion outliers, the six motion parameters, the mean white matter, and cerebrospinal fluid time series were fed into general linear models for nuisance regression to remove unwanted effects.

The region‐to‐region functional connectivity method was performed in this study to measure the correlations in spontaneous BOLD fluctuations. A network is comprised of nodes and edges; nodes being the brain region of interest (ROI) and edges being the connections between regions. Data are reported in 166 brain areas, as 5 regions in the 3D MRI Rat Brain Atlas were excluded from analysis due to the large size of three brains that fell slightly outside then field of view excluding signal from the most caudal tip of the cerebellum. Voxel time series data were averaged in each node based on the residual images using the nuisance regression procedure. Pearson's correlation coefficients across all pairs of nodes (14 535 pairs) were computed for each subject among all three groups to assess the interregional temporal correlations. The r‐values (ranging from −1 to 1) were *Z*‐transformed using the Fisher's *Z* transform to improve normality. 166 x 166 symmetric connectivity matrices were constructed with each entry representing the strength of edge. Group‐level analysis was performed to look at the functional connectivity in the experimental groups. The resulting *Z*‐score matrices from one‐group *t* tests were clustered using the K‐nearest neighbors clustering method to identify how nodes cluster together and form resting‐state networks. A *Z*‐score threshold of |*Z*| = 2.3 was applied to remove spurious or weak node connections for visualization purposes.

## 3 RESULTS

### 3.1 Voxel‐based morphometry

**FIGURE 1:**
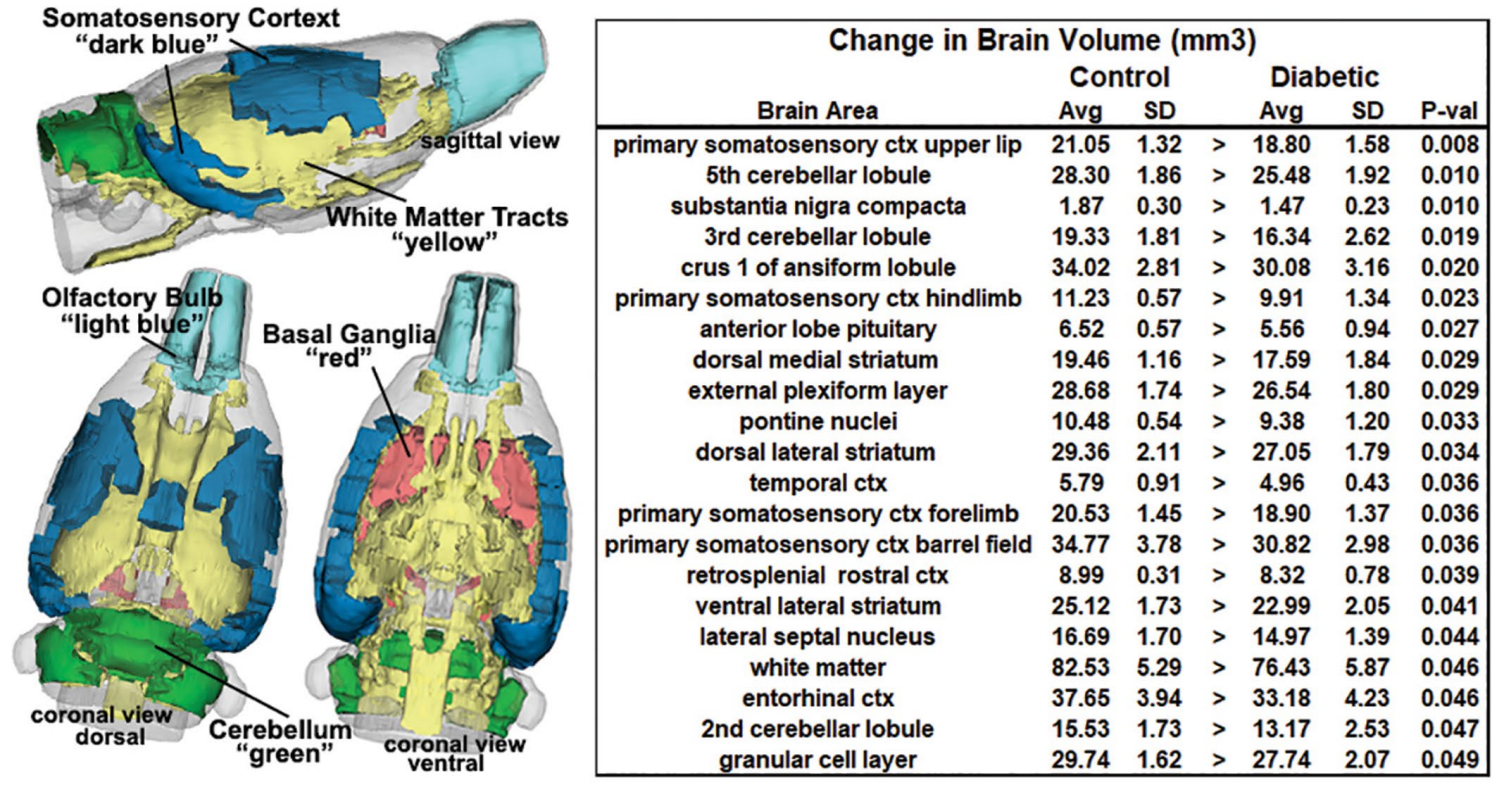
Voxel‐based morphometry. The table to the right list the brain areas that have significantly different volumes between experimental conditions. These brains areas are shown in the 3D reconstructions summarizing the differences

Figure 1 shows a table comparing the average brain volumes that were significantly different between control and BBZDR/Wor rats. The brain areas are ranked in order of their significance and are truncated from a larger list of 171 areas (see Table S1). With a false detection rate of *P* = .024, there are only six areas deemed significant, three from cerebellum and two from the somatosensory ctx. Note, in all cases the BBZDR/Wor brain volumes are smaller than controls. While not statistically significant, this is also true for most brain areas (136/171) as shown in Table S1. The left‐hand side of Figure 1 shows a 3D reconstruction summarizing the brains areas listed in the table. Several areas of the somatosensory cortex (upper lip, hindlimb, forelimb, barrel field, entorhinal, temporal, and retrosplenial cortices) showed a reduced volume as did the white matter tracts, basal ganglia (striatum, substantia nigra, septum), anterior cerebellum (2nd , 3rd, and 5th lobules), and olfactory bulbs (granular and external plexiform layers).

### 3.2 Diffusion weighted imaging

**FIGURE 2:**
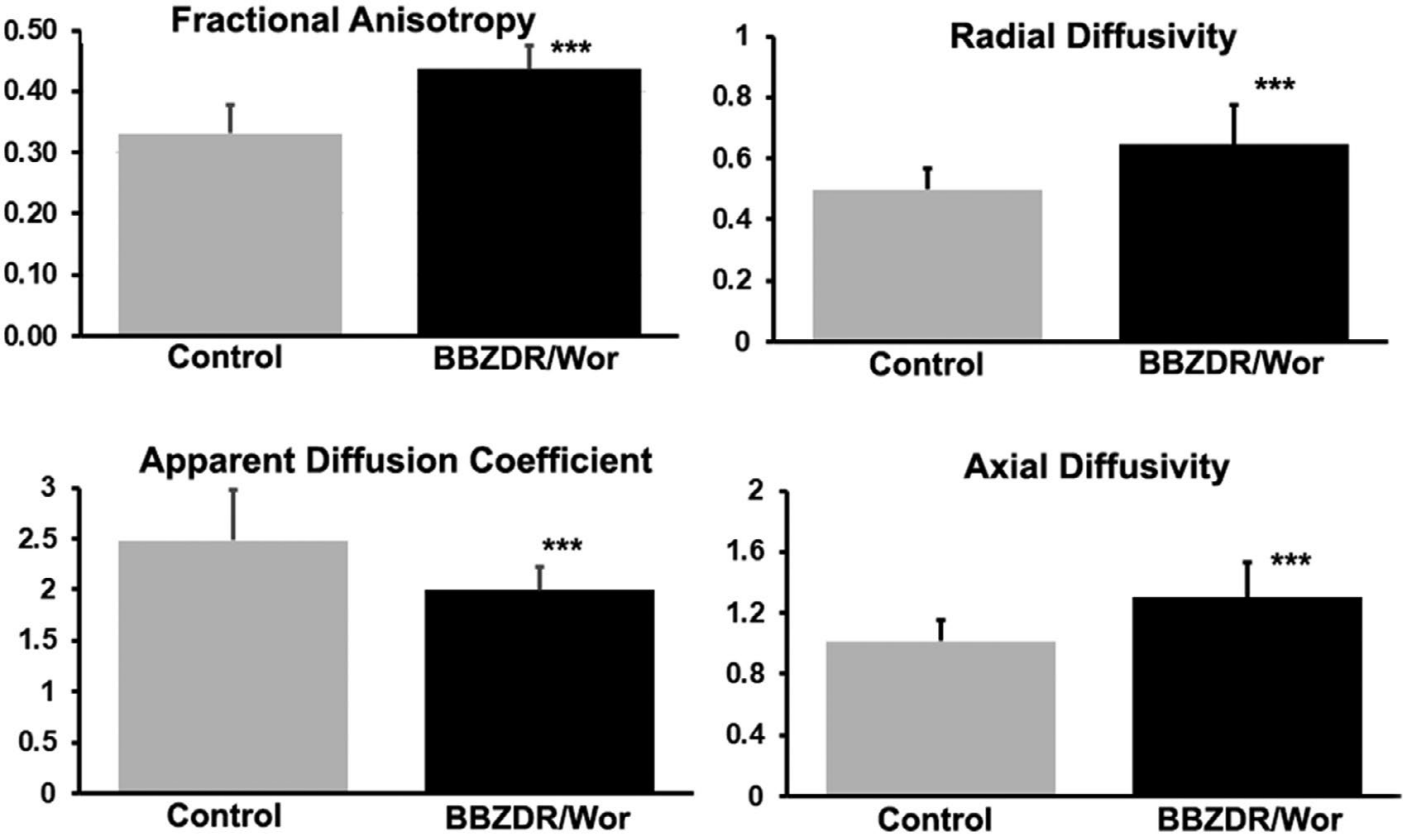
Diffusion weighted imaging. The bar graphs show the mean and standard deviation (vertical lines) for each index of anisotropy. ***P < .001

The significant differences in between control and BBZDR/Wor rats for ADC, FA, AD, and RD are presented in Figure 2. The mean and standard deviation for all significantly different brain areas for each index of anisotropy are shown. The values for FA, AD, and RD were significantly higher in BBZDR/Wor than control while just the opposite was true for ADC. Tables are provided in the Data S2‐S5 for each measure of anisotropy for all 171 brain areas ranked in order of their significance. In the case of ADC, 90/171 brain areas were significantly different; and in the case of FA, RD, and AD 111/171, 133/171, and 32/171 brain areas were significantly different.

### 3.3 Resting‐state functional connectivity

**FIGURE 3:**
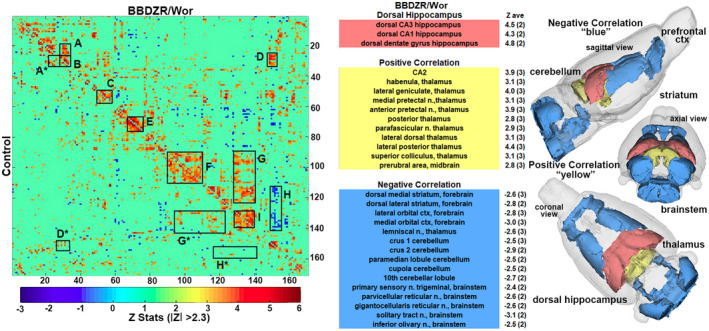
Resting‐sate functional connectivity. To the left is a correlation data matrix comparing control and BBZDR/Wor rats. To the right are tables of positively (yellow) and negatively (blue) coupled areas to the dorsal hippocampus (red) with 3D reconstruction summarizing the brain areas. A, intra‐thalamic connections; B, posterior thalamus; C, midbrain, D connections between the habenula/tectal/parafascicular thalamus and the dorsal hippocampus; E, prefrontal cortex; F, cerebellum/pons; G, connections between the cerebellum/pons and the trigeminal brainstem reticular activating system; H, hippocampus and the posterior cerebellum; and I is the brainstem reticular activating system and deep cerebellar nuclei

Figure 3 shows a correlation matrix comparing 166 brain areas for rsFC between the controls and BBZDR/Wor rats. Each colored red/orange pixel represents 1 of 166 brain areas that has a significant positive correlation with other brain areas. Pixels in shades of blue have a significant negative, or anticorrelation with other brain regions. The brain areas with significant correlations appear as clusters because they are contiguous in their neuroanatomy and function. The diagonal line separates the control and BBZDR/Wor groups. Each pixel on one side of the line has a mirror image pixel on the other side. The delineated areas serve to focus attention on similarities and differences in connectivity. Area A shows intrathalamic connections and favors greater coupling for BBZDR/Wor rats. Areas B and C show the posterior thalamus and midbrain. Area D shows connections between the habenula/tectal/parafascicular thalamus and the dorsal hippocampus with clear hypoconnectivity in BBZDR/Wor as compared to control. Area E highlights the area of the prefrontal cortex, and area F the cerebellum/pons. Area G shows a broad area of connections between the cerebellum/pons and the trigeminal brainstem reticular activating system again favoring higher coupling in BBZDR/Wor rats as compared to controls. Area I is the brainstem reticular activating system and deep cerebellar nuclei. Of interest is the anti‐correlation or uncoupling in area H (blue pixels) between the hippocampus and the posterior cerebellum. A table is provided showing the positive (yellow) and negative (blue) connections with the dorsal hippocampus (CA1, CA3, and dentate) highlighted in red. The *Z*‐score to the right of each brain area is the average score to the three areas of the dorsal hippocampus. For example, the lateral geniculate has significant connections to CA1, CA3, and dorsal dentate (denoted by the 3 shown in parentheses) the average of which is a *Z* score of 4. In contrast, the crus 2 of the cerebellum only has significant negative connections to two of the three hippocampal areas (denoted by two in parentheses) with an average *Z* score of negative 2.9. This relationship between hippocampus, thalamus, and cerebellum/brainstem is highlighted in 3D reconstructions to the right. The positive coupling between the dorsal hippocampus and multiple thalamic nuclei and the uncoupling or negative correlation to the dorsal striatum, posterior cerebellum, and brainstem are shown.

## 4 DISCUSSION

There are several rat models that may have translational benefits when using MRI to study T2DM.^1^ These include the GK rat,^33^ Zuker Diabetic Fatty (ZDF) rat,^55^ HFD/STZ rat,^48^ and BBZDR/Wor, to list a few. This exploratory study began the process of evaluating the BBZDR/Wor model of T2DM with multimodal imaging with the expectation of finding common neuroradiological measures of cerebral neuropathy as reported in the clinic. If so, the model would have the potential to provide valuable imaging data on disease progression from prediabetic to late stage diabetes and a noninvasive means of assessing the efficacy of therapeutic intervention on brain structure and function.

### 4.1 Voxel‐based morphometry

A reduction in brain volume is a consistent finding across all imaging studies in 2TDB.^4^ Cortical volumes show a decrease in gray ^11^, ^25^, ^32^, ^34^, ^38^ and white matter.^34^, ^38^ The underlying cause of the global reduction in brain volumes is unknown but is thought to be the consequence of small vessel disease.^30^ The general brain atrophy and reduction in gray matter is associated with diminished cognitive function.^11^, ^38^, ^54^, ^56^ Hippocampal volumes are smaller in T2DM vs age‐matched controls ^17^, ^38^, ^60^ and maybe a contributing factor to the cognitive decline. The BBZDR/Wor rat presented with several brain areas that were smaller as compared to age‐matched controls. These areas included the somatosensory, entorhinal, and temporal cortices but not CA1, CA3, dentate, and subiculum, the primary components of the hippocampal complex.

### 4.2 Diffusion weighted imaging

Diffusion weighted imaging is an indirect way of assessing the integrity of white and gray matter microarchitecture. A recently published study by Moghaddam and colleagues ^45^ provides a comprehensive review of the literature on T2DM and DWI. The literature is very consistent reporting changes in microarchitecture in the areas of the frontal‐temporal cortex, hippocampus, cerebellum, thalamus, and all of the major white matter tracts. The cognitive decline in T2DM is strongly associated with alterations in DWI in white matter tracts.^21^, ^42^, ^57^ With few exceptions, the general DWI profile in human T2DM is a decrease in FA and increase in ADC. This inverse relationship suggests a loss of microstructural integrity and network organization.^45^ The BBZDR/Wor rat also presented with global and pervasive changes in measures of DWI but opposite to that reported in humans. The FA and ADC values were inversely related with FA being greater than ADC a potential sign of global neuroinflammation and cytotoxic edema. The changes in DWI were extensive affecting much of the brain including the hippocampus, thalamus, amygdala, cerebellum, and white matter tracts.

### 4.3 Functional connectivity

A review by Macpherson and colleagues covers much of the literature on rsFC in T2DM.^35^ There is general agreement across multiple studies that T2DM presents with a reduction in connectivity in the default mode network, interconnections between the prefrontal cortex, parietal cortex, and hippocampus. Thalamic coupling to cortical and cerebellar regions is also reduced.^5^ The effect on global connectivity in this BBZDR/Wor diabetes model is contrary to that reported in the clinical literature. As shown in the connectivity matrix Figure 3, there is no clear reduction in connectivity when looking at the clusters highlighted. If anything, there are areas of the brain that show hyperconnectivity with diabetes. Intrathalamic connections are enhanced (area A), as are thalamic connections to the dorsal hippocampus (area D) and cerebellar/pontine connections to the brainstem reticular activating system (area G). The increase in connectivity is not unprecedented and can occur between some brain areas in human T2DM,^6^ but that would appear to be the exception. The hyperconnectivity observed in BBZDR/Wor maybe a compensatory response to underlying pathology as reported in traumatic brain injury^31^ or in young children with early T1DM.^44^

## 5 LIMITATIONS AND CONSIDERATIONS

As an exploratory study, we recognize its many limitations. (a) There were no female rats, an issue of concern given data showing differences in diabetic pathology between females and males.^19^, ^27^ Unfortunately, only males develop diabetes in the BBZDR/Wor strain of rats.^51^ (b) There were no measures of cognitive function to correlation with the MRI data as is routine with clinical studies. We were unable to collect behavioral data due to the severity of the obesity in the BBZDR/Wor rat. (c) We did not image for white matter hyperintensities, lesions associated with microvascular insult. Again, this is routine in clinical studies and would have aided in our analysis of BBZDR/Wor as a relevant imaging model for T2DM. (d) We were unable to use imaging, particularly DWI, to follow disease progression over the life of the animals. For example, the work of Gatto et al used different measures of anisotropy to follow disease progression in a mouse model of amyotrophic lateral sclerosis which enable them to identify changes in nervous tissue at presymptomatic stages of the disease ^16^ (e) Our rsFC studies were done under light isoflurane anesthesia. These studies could have been done under awake conditions as we have done so in many other task‐related BOLD imaging^22^, ^28^, ^58^ or phMRI studies^13^, ^15^ in rodents. However, “resting state” poses a dilemma in awake animal imaging no matter the level of acclimation prior to imaging.^14^ Any physical restraint will most likely have some level of stress; hence, the rsFC data were collected under light anesthesia. Nonetheless, numerous studies comparing the anesthetized and conscious states show similar rsFC data.^18^, ^24^

## 6 CONCLUSION

These limitations aside, the neuroradiological measures collected in BBZDR/Wor rats using multimodal imaging methods were not similar to those reported in T2DM patents. While the VBM data in BBZDR/Wor reflected the general findings in T2DM showing a decrease in specific brain areas and a global trend toward a reduction in volume, the hippocampus, a critical area linking changes with cognitive function to disease progression, was unaffected. The DWI changes in FA, ADC, and RD were global and pervasive with no specific areas that could be identified as potential biomarkers by which to follow the prediabetic to diabetic stages of cerebral neuropathy. The rsFC data were contrary to most findings in T2DM which report hypoconnectivity with disease progression. The hyperconnectivity in BBZDR/Wor rat most likely reflects an effort to compensate for the pathology that is not seen in humans. When these rats present with diabetes, they are morbidly obese, with a minimum of activity and responsivity to environmental stimuli. The inability to interrogate the BBZDR/Wor rat with a battery of behavioral and cognitive tests and correlate these changes with disease progression is another limitation in the model. From these observations, we would conclude the BBZDR/Wor rat is not an appropriate imaging model for T2DM.

## CONFLICT OF INTEREST

CFF has a financial interest in Animal Imaging Research, the company that makes the RF electronics and holders for animal imaging.

## AUTHOR CONTRIBUTIONS

All of authors have contributed substantially to the manuscript. Concept, drafting and interpretation–Ferris, Lawson, and Rentrup. Execution and analysis–Cai, Kulkarni, Lawson, and Rentrup.

## FUNDING INFORMATION

The financial support was provided in part by an HHMI‐funded Inclusive Excellence Award to Northeastern University, PI Ondrechen.

## Supporting information

Table S1 Table Brain Volumes

Table 1FA

Table 1ADC

Table 1AD

Table 1RD
